# Host-Targeting Agents to Prevent and Cure Hepatitis C Virus Infection

**DOI:** 10.3390/v7112898

**Published:** 2015-11-02

**Authors:** Mirjam B. Zeisel, Emilie Crouchet, Thomas F. Baumert, Catherine Schuster

**Affiliations:** 1 Inserm, U1110, Institut de Recherche sur les Maladies Virales et Hépatiques, 67000 Strasbourg, France; emilie.crouchet@etu.unistra.fr (E.C.); thomas.baumert@unistra.fr (T.F.B.); 2 Université de Strasbourg, 67000 Strasbourg, France; 3 Institut Hospitalo-Universitaire, Pôle Hépato-digestif, Hôpitaux Universitaires de Strasbourg, 67000 Strasbourg, France

**Keywords:** hepatitis C virus, host-targeting agent, direct-acting antiviral, viral resistance

## Abstract

Chronic hepatitis C virus (HCV) infection is a major cause of liver cirrhosis and hepatocellular carcinoma (HCC) which are leading indications of liver transplantation (LT). To date, there is no vaccine to prevent HCV infection and LT is invariably followed by infection of the liver graft. Within the past years, direct-acting antivirals (DAAs) have had a major impact on the management of chronic hepatitis C, which has become a curable disease in the majority of DAA-treated patients. In contrast to DAAs that target viral proteins, host-targeting agents (HTAs) interfere with cellular factors involved in the viral life cycle. By acting through a complementary mechanism of action and by exhibiting a generally higher barrier to resistance, HTAs offer a prospective option to prevent and treat viral resistance. Indeed, given their complementary mechanism of action, HTAs and DAAs can act in a synergistic manner to reduce viral loads. This review summarizes the different classes of HTAs against HCV infection that are in preclinical or clinical development and highlights their potential to prevent HCV infection, e.g., following LT, and to tailor combination treatments to cure chronic HCV infection.

## 1. Introduction

Hepatitis C virus (HCV) is a hepatotropic enveloped RNA virus of the *Flaviviridae* family. It is a highly variable virus that has been classified into six major genotypes [1]. Approximately 170 million individuals worldwide are infected by HCV. Chronically HCV-infected individuals are at risk for developing cirrhosis and hepatocellular carcinoma (HCC) which are major indications for liver transplantation (LT). There is no vaccine to prevent HCV infection and, until recently, antiviral therapy (based on pegylated (PEG) interferon (IFN) alpha and ribavirin) only enabled a cure for less than half of the patients, with strong differences in treatment outcome depending on the genotype. Within the past years, with the sequential approval of novel antivirals specifically targeting viral proteins (direct-acting antivirals (DAAs)), chronic hepatitis C has become a curable disease in the majority of treated patients and the most recent DAAs act in a pan-genotypic manner (reviewed in [2]). Several novel antivirals are in late-stage clinical development and will further broaden the therapeutic arsenal against HCV and enable the tailoring of combination treatments for distinct patient groups.

Antivirals can be classified into two distinct categories depending on whether they target viral proteins, *i.e.*, DAAs, or interfere with cellular factors involved in viral infection, *i.e.*, host-targeting agents (HTAs). For many years, HCV-host interactions have remained largely elusive due to the lack of robust model systems, and anti-HCV drug development has paralleled discoveries about the molecular mechanisms of viral replication. Indeed, the establishment of increasingly sophisticated model systems to study HCV infection (reviewed in [3,4,5]) has enabled researchers to characterize the molecular mechanisms underlying the HCV life cycle in great detail (reviewed in [6,7,8,9]) and to design a wide variety of antivirals targeting various steps of the viral replication cycle (reviewed in [8,10]). While several HTAs had been developed to characterize the HCV replication cycle and corroborate the importance of defined host factors for distinct steps of viral infection, others have been uncovered through functional screening approaches aimed at repurposing existing small molecules/approved drugs [11,12,13]. This review summarizes the different classes of HTAs against HCV infection and highlights their potential for antiviral therapy to prevent viral resistance.

## 2. HTAs to Inhibit Various Steps of the HCV Life Cycle

**Figure 1:**
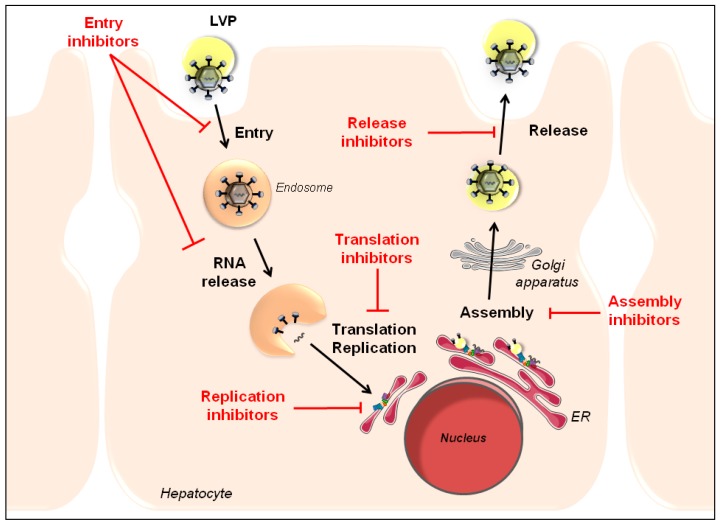
Schematic representation of the hepatitis C virus (HCV) life cycle and targets for antiviral therapy. HCV interacts with the basolateral membrane of hepatocytes, resulting in viral entry into the host cell. The virus is internalized *via* endocytosis and translation of the HCV RNA occurs in the cytoplasm following viral fusion and uncoating. Viral replication takes place within the cytoplasm in perinuclear endoplasmic reticulum (ER)-derived membranes called the “membranous web”. Progeny virions are assembled on cytosolic lipid droplets and subsequently transported along the secretory pathway and maturated in the Golgi before their release through microtubular transport and endocytic recycling compartment. Targets for antiviral therapy are highlighted in red.

The HCV life cycle can be subdivided into five different steps, including viral entry, viral translation, viral replication, viral assembly, and release of new virions. Each of these steps requires defined virus-host interactions to ultimately enable the virus to establish chronic infection. Given the amount of host factors involved in the HCV life cycle, this review solely focuses on some of these host factors that have been suggested to represent potential targets for HTAs during distinct steps of the HCV life cycle within hepatocytes (Figure 1). Other compounds exhibiting a broader mechanism of action by modulating the host’s immune responses, also termed biological response modifiers (Table 1), such as IFNs, Toll-like receptor (TLR) 7, or TLR9 agonists [14,15,16,17], are not reviewed in detail here.

**Table 1:** Stage of development of host-targeting agents (HTAs) for prevention and/or treatment of HCV infection. Only HTAs having at least reached *in vivo* preclinical development are listed.

Category	Target	Compounds	Stage of Development	References
**Entry inhibitors**	CD81	mAbs	Mouse model	[18]
	SR-BI	mAbs	Mouse model	[19,20,21]
		ITX-5061	Phase 1	[22,23]
	CLDN1	mAbs	Mouse model	[24]
	EGFR	Erlotinib	Phase 1/2	NCT01835938
	NPC1L1	Ezetimide	Mouse model	[25]
	Endocytosis/fusion	Silymarin/silibinin	Phase 2/3	[26,27]
		Chloroquine	Phase 4	NCT02058173
**Translation inhibitors**	miR-122	Miravirsen	Phase 2	[28,29]
**Replication inhibitors**	miR-122	Miravirsen	Phase 2	[28,29]
	HMGCoA reductase	Statins	Phase 3	[30,31]
	Cyclophilin	Alisporivir	Phase 2/3	[32]
		SCY-635	Phase 2	[33]
		NIM811	Phase 2	[34]
**Assembly inhibitors**	α-glucosidase 1	Celgosivir	Phase 2	[35]
	DGAT-1	LCQ908	Phase 2	NCT01387958
	Cyclophilin	NIM811	Phase 2	[34]
	PPARα	Naringenin	Phase 1	[36]
	HNF4α	Bezafibrate	Phase 4	[37]
**Biological response modifiers**	Immune responses	IFN-α	FDA-approved	www.hcvguidelines.org
		IFN-λ	Phase 3	NCT01795911
		TLR7 agonist	Phase 1	[15,16]
		TLR9 agonist	Phase 3	[17]
		Thymosin α1	Phase 3	[38]
		Nitazoxanide	Phase 2	[39,40]

mAbs: *monoclonal antibodies, EGFR: epidermal growth factor receptor;* IFN : interferon; TLR : Toll-like receptor; CLDN1: claudin 1; CD81 : cluster of differentiation 81; SR-BI: scavenger receptor class B type I; NPC1L1: Niemann-Pick C1-Like 1; HMGCoA: 3-hydroxy-3-methylglutaryl-coenzyme A; DGAT-1: diacylglycerol O-acyltransferase 1; PPARα : peroxisome proliferator-activated receptor alpha; HNF4α : hepatocyte nuclear factor 4 alpha. FDA: Food and Drug Administration; miR-122: microRNA-122

### 2.1 Entry Inhibitors to Prevent Initiation of Viral Infection and Viral Dissemination

**Figure 2:**
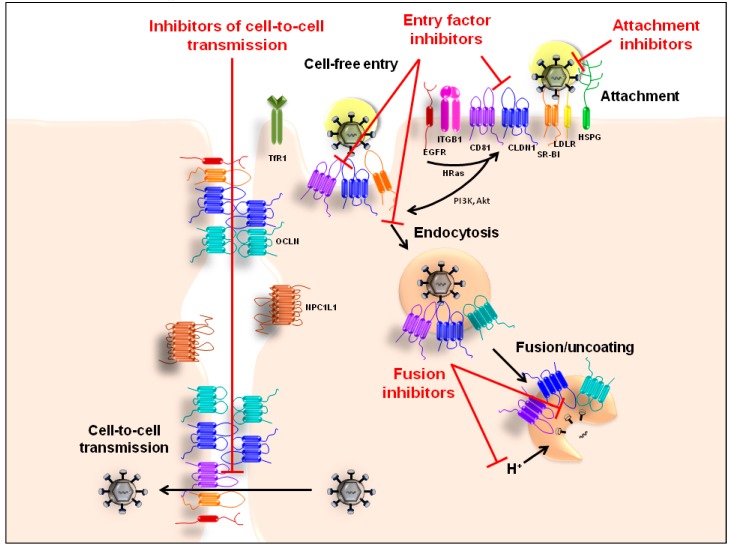
Schematic representation of HCV entry. The initial viral attachment on the basolateral membrane of hepatocytes is believed to involve the interaction of the viral particle—both viral (HCV envelope glycoproteins) and host-derived (apolipoproteins) factors—with HSPGs (heparan sulfate proteoglycans), LDLR (low density lipoprotein receptor) and SR-BI (scavenger receptor class B type I). Following interaction between the virus and different host factors expressed at the hepatocyte cell surface, including CD81 (cluster of differentiation 81) and CLDN1 (claudin 1), as well as rearrangement of cell surface proteins, the virus is ultimately internalized into its host cell *via* clathrin-mediated endocytosis. Additional host factors, including EGFR (epidermal growth factor receptor), NPC1L1 (Niemann-Pick C1-like 1) and TfR1 (transferrin receptor 1), contribute to the HCV entry process, for example by modulating intracellular signaling pathways or endocytosis. Following acidification of the endosome and subsequent fusion of viral and endosomal membranes, the viral genome is released into the cytoplasm. In addition to cell-free virus entry, HCV has also been described to transmit between hepatocytes through direct cell-to-cell transmission involving CD81, SR-BI, CLDN1, OCLN (occludin), EGFR, EphA2 (ephrin receptor A2) and NPC1L1. HTAs can interfere with different steps of the HCV entry process as highlighted in red.

The HCV entry process has been particularly well characterized within the past years (for a review see [6]). The initial viral attachment on the hepatocyte cell surface is believed to involve the interaction of the viral particle with heparan sulfate proteoglycans (HSPGs) [41,42,43,44,45,46], particularly with syndecan 1 (SDC1) [47] and syndecan 4 (SDC4) [48], low density lipoprotein receptor (LDLR) [49,50,51,52,53], and scavenger receptor class B type I (SR-BI) [54,55,56,57,58,59]. Interestingly, both viral (HCV envelope glycoproteins) and host-derived (apolipoproteins) factors within the viral particle appear to mediate this process (reviewed in [6,60]). Thus, the very first steps of viral interaction with the host cell surface can be inhibited by targeting host factors expressed either on the viral particle or on the host cell membrane (Figure 2). Indeed, it has been shown that synthetic anti-lipopolysaccharide peptides that bind to heparan sulfate moieties on the cell surface as well as antibodies directed against SR-BI or LDLR inhibit HCV attachment/infection [53,59,61]. Likewise, peptides that mimic the receptor binding domain and the HSPG binding domain of apolipoprotein E (apoE) inhibit HCV infection [45,48] and antibodies directed against apoE [45,62,63] as well as preincubation of recombinant cell culture-derived HCV (HCVcc) with soluble LDLR have also been shown to neutralize HCV infection, likely at the attachment/entry level [53,64]. Recently, it has been suggested that low-molecular-weight lignin, a component of *Lentinula edodes* mycelia solid culture extract, that has been reported to exhibit hepatoprotective activity, might inhibit HCV attachment through binding to apoE on the viral particle [65] given the structural similarity between lignin sulfate and heparan sulfate [66]. Interestingly, lipoprotein lipase (LPL) increases HCV attachment to the target cell by bridging virus-associated lipoproteins and cell surface heparan sulfate, whereas antibodies as well as a small molecule inhibitor-targeting LDLR have been shown to decrease HCV uptake [67,68]. In addition to its bridging function, LPL has been shown to inhibit viral entry by immobilizing the virus at the cell surface [64,69]. Most recently, it has been shown that very low-density lipoprotein (VLDL) is a serum component that inhibits HCV attachment [70].

Following initial attachment to the hepatocyte basolateral membrane, the virus interacts with additional host factors expressed at the hepatocyte cell surface to ultimately enter its host cell *via* clathrin-mediated endocytosis. It is believed that the interaction of the HCV envelope glycoproteins E1 and E2 with SR-BI, CD81, CLDN1 and potentially OCLN plays a major role in this process and some of these host factors have thus been suggested to play the role of “HCV receptors” [54,71,72,73,74,75]. Cluster of differentiation 63 (CD63) may also directly interact with HCV E2 and contribute to HCV entry [76]. Of note, additional host factors, termed “co-factors”, including various kinases, e.g., epidermal growth factor receptor (EGFR), ephrin receptor A2 (EphA2), AP-2-associated protein kinase 1 and cyclin G-associated kinase (GAK), as well as Niemann-Pick C1-like 1 (NPC1L1) and transferrin receptor 1 (TfR1), also contribute to the HCV entry process, for example by modulating intracellular signaling pathways or endocytosis [25,77,78,79]. Indeed, downstream EGFR signaling pathways including the GTPase Harvey rat sarcoma viral oncogene homolog (HRas) have been shown to be required for HCV entry, particularly for the formation of the CD81-CLDN1 co-receptor complex that is essential for this process (reviewed in [80]). Thus, HCV entry can be efficiently inhibited using antibodies, peptides or other molecules binding to SR-BI, CD81, CLDN1, EGFR and TfR1 as well as small molecule inhibitors of EGFR, EphA2, GAK, NPC1L1 and HRas [25,77,78,81,82,83,84,85,86,87,88,89,90,91,92,93,94,95,96] (Figure 2). Furthermore, host antiviral immune responses may also contribute to inhibiting HCV entry by interfering with the viral entry process as demonstrated by the inhibition of the EGFR kinase activity and, consequently, CD81-CLDN1 co-receptor formation *via* induction of IFN-α inducible protein 6 (IFI6), an IFN-stimulated gene (ISG) [97].

Following internalization *via* clathrin-mediated endocytosis, the viral genome is released into the cytoplasm after fusion of viral and endosomal membranes. In addition to some of the compounds described above, HCV endocytosis and/or fusion can be efficiently perturbed using amphipathic DNA polymers and small molecules including two anti-malaria drugs (chloroquine, ferroquine), arbidol and sylimarin/silibinin [98,99,100,101,102,103] (Figure 2). Of note, in addition to its interference with HCV entry, silymarin/silibinin-derived compounds and chloroquine have also been reported to interfere with viral replication [100,104,105].

In addition to cell-free virus entry, HCV has been shown to also directly infect neighboring hepatocytes via a process called cell-to-cell transmission [106,107,108]. In contrast to the cell-free HCV entry described above, HCV cell-to-cell transmission is resistant to most of the neutralizing antibodies described so far and is thus believed to play a major role in the maintenance of chronic infection. Although the detailed molecular mechanisms remain to be uncovered, it has been reported that the HCV envelope glycoproteins and apoE on the virus particle as well as CD81, SR-BI, CLDN1, OCLN, EGFR, EphA2 and NPC1L1 on the host cell play an important role for cell-to-cell transmission [19,24,25,77,92,106,107,108] (Figure 2). Of note, it has been described that HCV can circumvent CD81 and SR-BI for this process [108,109]. However, the potential consequences for antiviral therapy targeting entry factors remain unknown (also see Section 3 below). Notably, viral variants that appear not to require SR-BI for their *in vitro* infectivity remain sensitive to SR-BI-specific antibodies *in vivo*, as demonstrated using the human liver-chimeric uPA-SCID mouse model [20].

Host-targeting entry inhibitors (HTEIs) including antibodies directed against host entry factors or co-factors as well as small molecule inhibitors have proven to efficiently prevent/delay HCV infection in the human liver-chimeric uPA-SCID mouse model [18,24,25,99,110,111] and, thus, may represent a valuable strategy to prevent liver graft infection following LT. Furthermore, they may also contribute to treatment of chronic hepatitis C since an antibody targeting CLDN1 has recently been shown to cure chronic HCV infection in human liver-chimeric uPA-SCID mice in the absence of detectable toxicity [24]. Finally, their combination with other HTAs and/or DAAs can result in a synergistic antiviral activity [21], which provides perspectives for novel combination therapies for the cure of chronic hepatitis C (also see Section 3 below). Collectively, these data highlight the potential of HTEIs not only for prevention of HCV infection, e.g., during LT, but also for treatment of chronic hepatitis C. Of note, a phase 1/2 clinical trial evaluating erlotinib, a clinically licensed EGFR inhibitor used in cancer therapy, has been initiated in chronic HCV patients (https://clinicaltrials.gov/; Identifier: NCT01835938). Furthermore, silymarin/silibinin-derived compounds have been evaluated in phase 2/3 clinical trials and (case) reports suggest their efficacy in decreasing viral loads in chronic HCV patients and prevention of hepatitis C recurrence after LT, although other studies reported viral rebounds [26,112,113,114,115]. Whether this potential effect is (partially) due to silymarin/silibinin’s interference with viral entry remains to be defined. While the efficacy of chloroquine for treatment of non-response HCV patients is currently being evaluated in a phase 4 clinical trial (https://clinicaltrials.gov/; Identifier: NCT02058173), no clinical trial of ferroquine in hepatitis C patients has been registered so far.

### 2.2 Translation Inhibitors to Prevent Subsequent Viral Replication

Following viral endocytosis and fusion, HCV RNA is translated in an internal ribosome entry site (IRES)-mediated manner. The highly conserved IRES is located in the 5′-untranslated region (5′-UTR) of the viral genome [116,117]. HCV can thus directly recruit the translation initiation complex to the viral RNA to trigger viral protein translation *via* a cap-independent mechanism. The HCV IRES binding to the 40S ribosomal subunit is independent of canonical or non-canonical initiation factors. Moreover, HCV translation initiation only requires eukaryotic initiation factors (eIF) eIF2, eIF3 and eIF5 [118,119]. In contrast, optimal IRES activity is supported by the recruitment of several host proteins named IRES transacting factors (ITAFs) (for a review, see [7]), which represent a novel target for therapeutic intervention. Indeed, a synthetic peptide corresponding to the N-terminal 18 amino acids of the La antigen (efficiently blocks HCV translation by binding and sequestering other ITAFs [120].

IRES-ribosome interaction has been shown to be mediated through the binding of the 40S subunit to the stem loop III, domain e and f (SLIIIef), of the HCV IRES. A recent study demonstrated that a small RNA mimicking the SLIIIef domain (SLRef) selectively inhibits HCV translation by retaining the ability to interact with the 40S subunit. Interestingly, in line with previous studies, it was shown that the anti-HCV activity of SLRef could be mainly due to interaction and sequestration of the La antigen [121]. Ribosome-HCV IRES association can also be disturbed by prostaglandin A1 (PGA1) which exhibits dose-dependent inhibitory effects on HCV translation [122]. Indeed, its antiviral effect occurs through PGA1/eIF3-40S subunit/HCV IRES RNA complex formation which inhibits HCV IRES-mediated translation. Furthermore, repetitive PGA1 treatment appears to be safe in cell culture [122]. The *in vivo* efficacy of molecules interfering with HCV IRES-ribosome association remains to be determined.

Furthermore, microRNA-122 (miR-122) plays an important role in HCV translation. MiR-122, one of the most abundant liver-expressed miRNAs, binds to the HCV genome and enhances viral translation and replication [123,124]. Sequestering miR-122 using miravirsen, a locked nucleic acid-modified oligonucleotide complementary to the 5′-end of miR-122, showed prolonged and dose-dependent reduction in HCV viremia in patients without evidence of long-term safety issues [28,29] (also see Section 2.4 below).

Recently, the receptor for activated C kinase 1 (RACK1), a component of the ribosomal 40S subunit [125,126], was identified as an essential factor for HCV IRES-mediated translation [127]. Interestingly, RACK1 is not required for the classical cap-dependent translation and inhibition of RACK1 expression in cell culture models did not reveal major toxicity [127]. Thus, the development of RACK1 inhibitors may be an attractive perspective for future anti-HCV therapy.

### 2.3 Replication Inhibitors to Inhibit Viral Genome Amplification

Following translation and cleavage of the HCV polyprotein, the viral replicase complex, composed of HCV non-structural (NS) proteins, NS3, NS4A, NS4B, NS5A and NS5B, is assembled. HCV replication is a highly coordinated process involving host factors in addition to the replicase complex. Several functional small interfering RNA (siRNA) and chemical screens have been performed to uncover host factors involved in HCV replication and have provided useful insights into this process [128,129,130,131,132,133,134,135].

HCV has been demonstrated to remodel intracellular membranes, including endoplasmic reticulum (ER) membranes, for its replication and this process takes place at the so-called “membranous web” [136]. Indeed, HCV NS proteins induce the formation of double-membrane vesicles [136,137,138,139]. It has been suggested that this process may be linked to the activation of the autophagy pathway [140], a cellular catabolic process where protein aggregates and damaged organelles are removed for recycling. Indeed, several studies suggested that HCV activates the autophagic pathway to support its replication (reviewed in [141,142]). Moreover, HCV replication has been reported to occur on autophagosomal membranes [140,143]. This may enable the virus to conceal its RNA and evade double-stranded RNA-triggered host antiviral responses [140]. Chloroquine has been described as an inhibitor of autophagic protein degradation and, in addition to inhibiting HCV entry by modulating endosomal acidification [98], chloroquine has been shown to reduce HCV replication [105]. Host proteins have also been reported to contribute to the formation of double-membrane vesicles (reviewed in [144]), e.g., cyclophilin A (CypA), which interacts with NS5A, and proline-serine-threonine phosphatase interacting protein 2 (PSTPIP2), which interacts with NS4B and NS5A [145,146]. The importance of CypA for the HCV life cycle has recently been underscored in a humanized mouse model of HCV infection in which the *Ppia* gene had been knocked out [147]. In addition to CypA, several other cyclophilins (Cyps) have been reported to also contribute to HCV replication [128,148,149,150]. Cyclosporines are classical Cyp inhibitors; however, their immunosuppressive properties preclude their use as antivirals. Within the past years, several alternative Cyp inhibitors have been generated: cyclosporine A (CsA) derivatives without immunosuppressive properties and new molecules structurally unrelated to CsA (reviewed in [151]). Cyp inhibitors were the first class of HTAs against HCV reaching clinical development. Three non-immunosuppressive Cyp inhibitors have so far demonstrated clinical efficacy in chronic hepatitis C patients in IFN-based as well as IFN-free treatment regimens [32,151]. These Cyp inhibitors have been shown to disrupt/prevent the formation of CypA-NS5A complexes and thereby inhibit HCV replication [33,151,152,153,154]. In addition, the anti-HCV activity of CypA inhibitors may be increased since they appear to restore the host innate immune responses to HCV [33].

NS5A is a central player within the membranous web as it interacts with a plethora of host factors, thereby orchestrating viral replication. The lipid kinase phosphatidyl-inositol-4-kinase III alpha (PI4KIIIα) is one of the major NS5A partners in this process [155]. Indeed, NS5A binds to and activates PI4KIIIα and this, in turn, regulates the phosphorylation status of NS5A [156]. PI4KIIIα activity leads to phosphatidyl-inositol-4-phosphate (PI4P) accumulation within the membranous web and likely the subsequent recruitment of cellular lipid transporters leading to the modulation of its lipid content (reviewed in [144]). In the absence of PI4KIIIα activity or through alteration of its interaction with NS5A, changes in the ultrastructural morphology of the membranous HCV replication complex were observed [132,155,156]. PI4KIIIβ has also been reported to contribute to the replication of HCV genotype 1 but not genotype 2 [157,158]. PI4KIII inhibitors able to reduce HCV replication *in vitro* have been developed, but their *in vivo* toxicity as well as the lethal phenotype of transgenic mice exhibiting kinase-defective PI4KIIIα preclude their further clinical development as antivirals [134,158,159,160,161]. As stated above, the host cell lipid metabolism plays an important role in the HCV replication complex [162]. Particularly elements of cholesterol and fatty acid synthesis and geranylgeranylation of host proteins have been shown to modulate viral replication. Indeed, several genes involved in lipid metabolism have been reported to be modulated during acute HCV infection of chimpanzees [163]. HCV replication can be disrupted *in vitro* by treatment with inhibitors of 3-hydroxy-3-methylglutaryl CoA (HMGCoA) reductase or with an inhibitor of protein geranylgeranyl transferase I [164,165]. Notably, not all HMGCoA reductase inhibitors affect HCV replication, suggesting the existence of a different structure-activity relationship between the anti-HMGCoA reductase activity and the antiviral activity [166]. Fluvastatin is a statin that has been reported to increase the efficacy of IFN-based treatment in chronic HCV-infected patients [30,31,167,168]. Recent *in vitro* studies have provided novel insights into the mechanism of action of fluvastatin. The anti-HCV effect of fluvastatin has been suggested to be due to the induction of microtubule bundling and a decrease of expression of the microtubule binding partner doublecortin-like kinase 1 (DCLK1) [169]. Furthermore, fluvastatin and other statins exhibiting an antiviral effect against HCV induce the expression of heme oxygenase-1 (HO-1), which has been reported to reduce HCV replication [170,171,172,173]. HMGCoA reductase activity can also be inhibited using SKI-1/S1P inhibitors [174,175]. In addition to inhibiting HCV replication, SKI-1/S1P inhibitors may also reduce the production of new virions [174]. Interestingly, it has also been reported that antibody-mediated blocking of the LDLR leads to an increase in the ratio of phosphatidylethanolamine to phosphatidylcholine in host cells and a decrease in HCV replication [64], further strengthening the link between lipid metabolism and HCV replication. Moreover, a functional chemical compound screen identified the estrogen receptor alpha (ESRα) as an interaction partner of NS5B within lipid rafts on ER membranes [176]. Indeed, tamoxifen, a partial ESR agonist and prototype selective estrogen-receptor modulator, as well as a decoy peptide against ESRα-NS5B interaction inhibited HCV replication and ESRα-NS5B interaction in the replicase complex [176]. ESRα may thus also represent a potential target for anti-HCV therapy. Finally, it has been recently reported that HCV replication can also be inhibited by cholesterol-25-hydroxylase (CH25H) whose expression is increased upon HCV infection [177,178,179]. CH25H synthesizes 25-hydroxycholesterol (25HC) which displays antiviral activities against different enveloped viruses and may thus represent a broad-spectrum antiviral [163,180]. The anti-HCV activity of CH25H appears to be mediated through 25HC-dependent and independent events including the interaction with NS5A that prevents NS5A dimerization required for viral replication, the inhibition of the formation of the membranous web, and the disruption of the function of the transcription factors sterol regulatory element-binding proteins (SREBPs), thereby perturbing the host lipid metabolism [177,179].

In addition to its role in HCV genome translation as described above, miR-122 is a hepatocyte-specific host factor essential for HCV replication. Indeed, HCV cannot replicate in cells lacking miR-122 or in which miR-122 has been sequestered [123,181,182,183,184,185]. The viral RNA contains two adjacent miR-122 sites in its 5′ UTR and miR-122 binding to the HCV genome promotes viral replication [186,187]. MiR-122 thus represents an interesting target for antiviral therapy (reviewed in [188]) and the miR-122 inhibitor miravirsen has proven its efficacy in reducing viral loads in chronic hepatitis C patients in phase 1/2 clinical trials [28,29]. The antiviral activity of miravirsen may be due to two complementary mechanisms: the hybridizing to mature miR-122 that blocks its interaction with HCV RNA, and the binding to the stem-loop structure of pri- and pre-miR-122 that inhibits processing of miR-122 precursors [189].

Besides inhibiting host factors required for viral replication, HCV replication may also be inhibited by modulating host factors that restrict HCV replication, e.g., the elongation factor Tu GTP binding domain containing 2 (EFTUD2) and the human cytidine deaminase APOBEC3G (hA3G) [190,191,192]. Interestingly, hA3G stabilizers exhibit antiviral activity against HCV as hA3G binds to NS3, thereby inhibiting its helicase activity required for viral replication [190,193]. Given their original mechanism of action, this class of HTAs may deserve further research to optimize their activity.

Interestingly, compounds inhibiting histone deacetylase 6 (HDAC6) have also been reported to inhibit HCV replication [194,195]. The mechanism of action appears to involve hyperacetylation of α-tubulin [194]. Finally, a signal transducer and activator of transcription 3 (STAT3) inhibitor with anti-HCV effect has recently been uncovered in a high-throughput screen [135]. Indeed, STAT3 has been reported to be activated by HCV core and NS5A and to act as a proviral factor for HCV replication [196,197,198].

### 2.4 Assembly/Release Inhibitors to Prevent Generation and Release of New Virions

**Figure 3:**
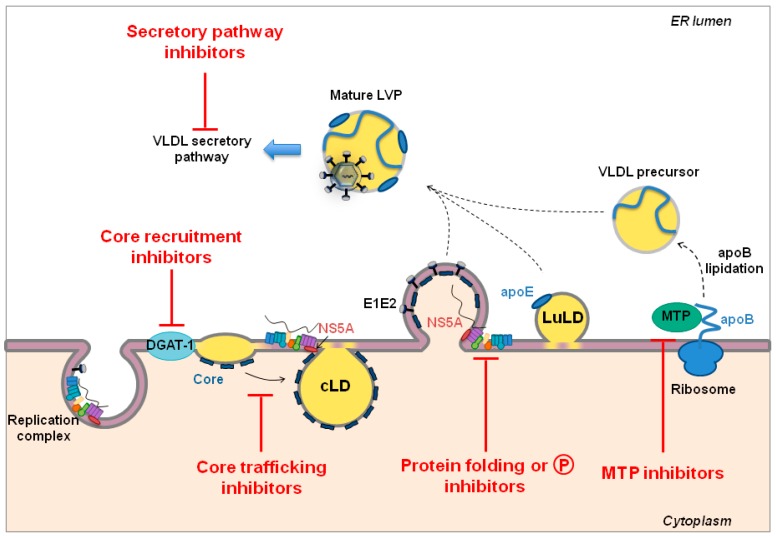
Schematic representation of HCV assembly. Virion assembly is triggered by core recruitment on cytosolic lipid droplets (cLDs) by the diacylglycerol acyltransferase-1 (DGAT-1). In turn, replication complexes are recruited through core-NS5A interactions. Nucleocapsid formation is mediated by viral budding into the ER lumen, at the site of VLDL production. The immature viral particles fuse or attach to a luminal LD (LuLD) through apoE-E1E2 and apoE-NS5A interactions. During VLDL synthesis, apolipoprotein B (apoB) is directly produced in the ER lumen and enriched in lipids by the microsomal triglyceride transfer protein (MTP) to generate VLDL precursors. Viral particles merge with these nascent VLDLs to generate mature lipoviroparticles (LVPs). LVPs enter the VLDL maturation and secretion pathway to be released from hepatocytes. HCV assembly and egress can be impaired by targeting different steps of these processes as highlighted in red.

A remarkable hallmark of HCV is its association with VLDL/low density lipoprotein (LDL) to form an infectious lipoviroparticle (LVP) [199]. Intracellular assembly and egress of LVP depend on numerous factors involved in lipid metabolism (reviewed in [162,200]) and are closely linked to the VLDL production machinery [201]. Among the main factors involved in this process are lipid droplets (LDs), intracellular organelles that originate from triglyceride (TG) and cholesterol ester (CE) accumulation between the two leaflets of the ER bilayer [202]. This microenvironment represents the “platform” for HCV particle assembly. Several host factors involved in TG biosynthesis and LD biogenesis have been demonstrated to contribute to this process and thus represent targets for HTAs (Figure 3). Indeed, HCV morphogenesis is triggered by the recruitment of the viral core protein from the ER to LDs [203], a process mediated by the diacylglycerol acyltransferase-1 (DGAT-1) (Figure 3). Interestingly, a DGAT-1 chemical inhibitor, currently in clinical trials for metabolic diseases (reviewed in [204]), might serve as an antiviral against HCV infection [205]. Notably, since in hepatocytes DGAT-2 has redundant functions in LD morphogenesis, this DGAT-1-specific inhibitor did not impair LD homeostasis *in vitro* [205]. The safety of DGAT-1 inhibitor LCQ908 has already been evaluated in HCV-infected patients (https://clinicaltrials.gov/; Identifier: NCT01387958), but additional studies are needed to assess its antiviral efficacy in patients.

Core recruitment to LDs also depends on the composition and the fluidity of ER lipid bilayers that is regulated by the cytosolic phospholipase A2, group 4A, (PLA2G4A) and upstream mitogen-activated protein kinases-extracellular signal regulated kinases (MAPK/ERK). PLA2G4A has been shown to contribute to HCV assembly since pyrrolidine-2 (Py-2), a chemical inhibitor of PLA2G4A, induces aberrant HCV particle production [206]. Furthermore, selective inhibitors of the MAPK/ERK pathway can block infectious HCV production [206,207]. However, opposite studies demonstrated that inhibition of the Ras/Raf/MEK/ERK pathway enhances HCV translation/replication, and disturbs IFN signaling [208,209,210]. The potential of MAPK inhibitors as antivirals thus remains to be further investigated. The cytosolic phospholipase A2, group 4C, (PLA2G4C) is another enzyme involved in LD homeostasis that can be targeted by HTAs to inhibit assembly. Indeed, the “methyl arachidonyl fluorophosphonate” molecule (MAFP) significantly reduced HCV assembly by sequestering viral proteins to the ER prior to virion assembly [211].

Directly targeting core trafficking in hepatocytes also represents an interesting antiviral strategy given the numerous host factors involved in this process (Figure 3). These include AP2M1, the μ subunit of the clathrin adaptor protein complex 2 (AP-2) that interacts with the core protein through a process involving the adaptor-associated kinase 1 (AAK1) and the cyclin-associated kinase (GAK) [79,96,212]. Approved anticancer kinase inhibitors, such as erlotinib or dasatinib targeting GAK and sunitinib or PKC-412 targeting AAK1, can disrupt core-AP2M1 interaction and, thus, HCV assembly [79,96,213]. However, it has to be pointed out that these compounds were initially designed to target other kinases—some of which are involved in HCV entry (see Section 2.1 above)—and thus lack selectivity for GAK and AAKI. The recently reported GAK inhibitor isothiazolo[5,4-b]pyridine that displays limited off-target binding to other kinases efficiently inhibits HCV entry and assembly, and may thus be further developed as a potential antiviral [96].

Other HTAs interfering with host cell lipid metabolism have been reported to display antiviral activity against HCV. Indeed, resveratrol that increases the expression of PPARα, a nuclear transcription factor that activates fatty acid oxidation and is decreased by the core protein [214,215], impairs HCV assembly and secretion by enhancing consumption of fatty acids [216]. Moreover, recent screens identified pterostilbene, a methylated form of resveratrol, toremifene, an estrogen receptor modulator similar to tamoxifen, and quinidine, an antiarrhythmic drug, as potential antivirals against HCV [11,12]. Naringenin was also shown to block production of infectious HCV particles through the activation of PPARα [36]. Interestingly, HCV can also repress PPARα activity and lipid accumulation by inducing up-regulation of miR-27b and bezafibrate, a PPARα agonist, which reverses miR-27b-induced lipid accumulation [217]. This indicates the potential of additional strategies to design novel HTAs against HCV.

Besides core, NS5A is another major player within the HCV replication cycle that connects replication and assembly. NS5A-core interaction on LDs is a crucial step for viral assembly [203,218,219,220]. Assembly competence of NS5A is mediated by the Caseine Kinase (CK)-mediated phosphorylation of multiple serine and threonine residues [221,222]. Indeed, the specific CKII inhibitor, 2-dimethylamino-4,5,6,7-tetrabromo-1H-benzimidazole (DMAT), was shown to dramatically disrupt virion biogenesis [221] (Figure 3). However, HCV genotype 1a virus production did not appear to be affected by DMAT [223]. Thus, genotype-specific differences might have to be taken into account for potential future clinical application of such inhibitors. To date, one chemical inhibitor of CKII, CX-4945, has entered clinical trials for its antitumor activity, but the potential antiviral effect against HCV remains to be determined [224].

HCV particle assembly and egress appear to share several host factors with VLDL assembly and secretion pathways [201,225] (Figure 3). The early step of VLDL synthesis consists of lipidation of the newly synthetized apolipoprotein B (apoB) across the ER membrane by the microsomal triglyceride transfer protein (MTP). In the second step, the VLDL precursors are enriched in lipids *via* fusion with resident apoE containing LDs (for a review, see [226]). Accumulating evidence demonstrates the crucial role of apoE in HCV assembly [62,63,227,228,229,230,231], while the role of apoB and MTP still remains unclear and controversial [62,63,201,225,232]. Nevertheless, a recent study demonstrated the ability of amiodarone, a cationic amphipathic drug that inhibits MTP activity, to down-regulate HCV assembly and release in cell culture [233] (Figure 3). Several other MTP inhibitors are in clinical trials for the treatment of hyperlipidemia (reviewed in [234]). However, whether amiodarone and other MTP inhibitors display an antiviral effect against HCV *in vivo* remains to be determined.

The VLDL secretory pathway is also hijacked to facilitate HCV egress from infected hepatocytes [201,225] (Figure 3). VLDL secretion is regulated by the hepatocyte nuclear factor 4α (HNF4α), the most abundant transcription factor in the liver. Interestingly, HNF4α also regulates expression of phospholipase A2 GXIIB (PLA2GXIIB), a crucial host factor for HCV production [235]. HNF4α down-regulation by bezafibrate dramatically reduced HCV secretion [235]. In line with this study, a meta-analysis showed that bezafibrate is associated with a reduction of viral load in chronically HCV-infected patients [168]. Moreover, in association with IFN and ribavirin, bezafibrate has been reported to reduce serum HCV RNA titers and to improve liver dysfunction [37]. Cyps have also been reported to contribute to HCV virion secretion in addition to their role in viral replication [150]. The Cyp inhibitor NIM811 alters lipid trafficking and decreases apoB secretion through the VLDL pathway, thereby blocking HCV production [236]. The combination of NIM811 with PEG-IFNα showed significant antiviral activity compared to IFN alone in chronically infected HCV patients [34]. This synergistic effect may be due to the inhibition of both HCV replication and assembly by NIM811.

To counteract HCV proliferation, several studies reported a novel antiviral strategy that uses the human heat shock cognate protein 70 (Hsc70) as an antiviral target [237,238,239,240]. Hsc70 was identified as a part of the viral particle that modulates virion capsid assembly and LD-dependent virus release [241]. IMB-DM122 [237] and N-substituted benzyl matrinic acid [239] inhibit Hsc70 expression through destabilization of Hsc70 mRNA. Treatment of HCV-infected hepatocytes with these components reduced the incorporation of Hsc70 into viral nucleocapsids, and therefore disrupted HCV production. Moreover, both compounds demonstrated good oral pharmacokinetic profiles and high safety *in vivo*, making these molecules promising candidates for clinical trials [237,239]. Recently, it has been reported that (+)-lycoricidine exhibits anti-HCV activity through its inhibitory effect on the expression of Hsc70 [238]. However, the significant cytotoxicity of this compound precluded further development and new phenanthridines with reduced toxicity have been designed [240]. Their potential as antivirals remains to be determined.

Finally, a promising strategy developed to inhibit virion assembly is to interfere with glucosidases that ensure N-glycosylation and folding of HCV envelope glycoproteins E1 and E2 [242] (Figure 3). It was shown that celgosivir, an α-glucosidase I inhibitor, leads to reduced viral production and infectivity *in vitro*. Moreover, celgosivir administration in combination with PEG-IFN α and ribavirin displayed a clinical benefit in genotype 1 chronically infected patients (reviewed in [35]). Long-term studies are needed to confirm its safety in humans.

## 3. HTAs to Prevent HCV Resistance

**Figure 4:**
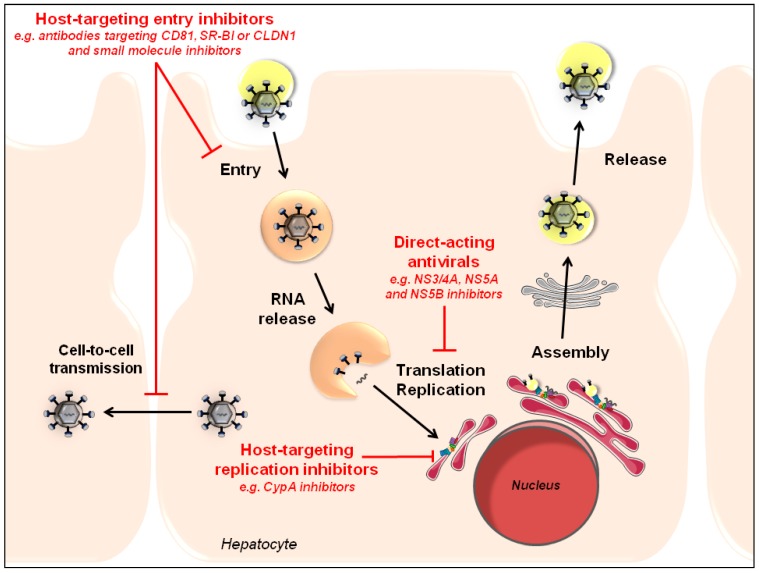
Synergy between HTAs and DAAs to inhibit HCV infection at different steps of the viral life cycle. Given their complementary mechanism of action, HTAs and DAAs can act in a synergistic manner to reduce viral loads and to prevent viral resistance. Different classes of HTAs and DAAs that have been evaluated in combination are highlighted in red.

Given that they target a highly variable virus, the main limitation of DAAs is viral resistance. Viral resistance may be prevented using different strategies including (i) targeting conserved viral sequences less prone to mutation; (ii) using a combination of drugs exhibiting distinct targets and mechanisms of action; and (iii) increasing host antiviral responses. In contrast to the first DAAs, the emergence of viral resistance with recently approved DAAs, such as sofosbuvir, is less frequent but has nevertheless been described [2,243,244,245]. Given the generally lower barrier to resistance of DAAs as compared to HTAs, the emergence of treatment-resistant viral variants during DAA therapy remains a challenge. By acting through a complementary mechanism of action and by exhibiting a generally higher barrier to resistance, HTAs can be associated with DAAs to prevent viral resistance. Indeed, given their complementary mechanism of action, HTAs and DAAs can act in a synergistic manner to reduce viral loads [13,21,103] (Figure 4). Interestingly, various HTAs may also be combined to synergistically inhibit HCV infection, as demonstrated for combinations of a HTEI and host-targeting replication inhibitor, *i.e.*, a Cyp inhibitor [21]. Furthermore, HTEIs have been demonstrated to prolong viral suppression by DAAs and to prevent viral dissemination of DAA-resistant strains *in vitro* [21,246,247]. Indeed, although treatment of HCV-infected cells with DAAs rapidly results in a dramatic reduction of viral load *in vitro*, viral rebound is usually observed following withdrawal of the drug. However, the addition of an HTEI to the cell culture at the time of DAA withdrawal allows the further decrease of the viral load, indicating that HTEIs limit viral rebound from DAA therapy [21,246]. Furthermore, as many HTEIs inhibit cell-to-cell transmission that has been shown to represent the major route of transmission of wild type and DAA-resistant viruses, HTEIs have been demonstrated to limit dissemination of DAA-resistant strains and consequently prevent antiviral resistance [247]. In line with these *in vitro* data, the Cyp inhibitor alisporivir, currently in phase 2/3 clinical trials, has been shown to efficiently reduce viral loads in chronic hepatitis C patients in combination with ribavirin [32] and future clinical trials evaluating its antiviral effect in combination with DAAs will provide further insights into the potential of this HTA.

Thus far, no viral resistance to HTAs was observed in preclinical or clinical studies [24,28,248]. However, some resistant viral variants could be selected by viral passaging *in vitro* [75,103]. This has been first evidenced and most extensively studied with Cyp inhibitors [249,250,251,252]. Of note, it was difficult to generate variants exhibiting Cyp inhibitor resistance and the time required to select resistance against these HTAs was significantly longer as compared to DAAs; furthermore, the resistance level was lower [249,250]. These data suggested that these viral variants were less dependent on CypA-dependent isomerization of NS5A for their replication [250]. Moreover, while prolonged administration of miravirsen (a miR-122 inhibitor) did not result in the emergence of viral variants exhibiting mutations within the miR-122 binding regions in preclinical and clinical trials [28,248], viral variants exhibiting a single nucleotide change in the 5′UTR between the miR-122 binding sites and requiring less miR-122 for their replication could be identified *in vitro* [253]. Whether the emergence of such variants could be avoided by combining miravirsen with other HTAs and/or DAAs with anti-HCV activity remains to be determined. Collectively, these studies indicate that HCV may become less dependent on defined host factors under selection pressure *in vitro*. While most mutants were still dependent on Cyp and/or miR-122, specific mutations in NS5A eliminated the dependence of HCV RNA replication on the expression of host CypA [152]. Notably, a recent study reported that HCV is able to change its host factor dependency under selection pressure. Indeed, in CLDN1 knock-out cells, a CLDN1-dependent strain was able to evolve to use CLDN6 or CLDN9 as a receptor [75]. Whether this may occur *in vivo* where CLDN1 is expressed at much higher levels than CLDN6 and CLDN9 remains to be determined (reviewed in [254]).

## 4. Conclusions and Perspectives

During the last decade, the development of new DAAs considerably improved anti-HCV therapy, displaying high cure rates in treated patients. However, treatment failure and resistance in a subset of patients remain a challenge. Alternative approaches targeting host factors involved in HCV pathogenesis offer new treatment opportunities. Due to low genetic variability of host factors, HTAs exhibit a high genetic barrier to resistance and display a pan-genotypic antiviral activity.

Several HTAs are currently in development in preclinical or clinical trials (Table 1). Proof-of-concept studies on alisporivir and miravirsen have already shown very high cure rates in phase 2 and 3 clinical trials when used in monotherapy. Preclinical studies have also demonstrated the potential synergistic activity of HTA/DAA combinations to cure HCV infection (Figure 4). Synergy between DAAs and HTAs offers a prospective option for combination therapies for treatment failure to licensed therapies. Furthermore, entry inhibitors, such as anti-receptor antibodies, could offer prevention for HCV graft infection during LT. Indeed, it has been shown that such inhibitors impair cell infection of highly variable quasispecies isolated from individual patients as well as escape variants resistant to host neutralizing antibodies [25,77,78,81,82,83,84,85,86,87,88,89,90,91,92,93,94,95,96]. However, their clinical efficacy remains to be demonstrated. To conclude, HTAs may widen the therapeutic arsenal against chronic HCV infection and simplify HCV therapy.
